# Gender Differences in the Performance of Language Assessment Scale Trivandrum (LEST) in Children Aged Between Zero and Three Years

**DOI:** 10.7759/cureus.63786

**Published:** 2024-07-03

**Authors:** Priyadarshini Ponvannan, Balaji Chinnasami, S Vaishali, Monitha Pinnamaneni, Subash S

**Affiliations:** 1 Department of Paediatrics, SRM Medical College Hospital and Research Centre, SRM Institute of Science and Technology, Chennai, IND

**Keywords:** covid-19, covid-19 vs. language development, speech delay in boys, language milestones, development, speech delay

## Abstract

Background

The Language Assessment Scale Trivandrum (LEST) is a commonly used scale to assess the language development of children aged between zero and three years. The scale is commonly utilized in healthcare and community environments; however, there are no publicly available gender-specific standards that are used in the scale. The current study set out to examine gender disparities observed in the test and determine whether future accurate assessments will require the creation of separate LEST scales for boys and girls.

Methodology

A cross-sectional study was conducted among 198 children aged between zero and three years, with 99 girls and 99 boys. Parents of all eligible children after obtaining consent were interviewed, and the LEST scale was used to assess them in the form of a questionnaire. The LEST scale has 33 test items, which are used to test language development.

Results

There were substantial gender disparities between girls and boys. Boys had a delay in acquiring language milestones compared to girls, and the difference was significant. Overall, 27 girls out of 78 delayed children (34.6%) and 51 boys out of 78 delayed children (65.4%) had language delays with a significant p-value of 0.003.

Conclusions

Our study suggests that boys follow a different timeline for achieving language milestones compared to girls. These findings need to be validated with a larger study, and if found to have a significant difference, separate scales can be developed for boys and girls to assess language-acquiring skills.

## Introduction

Language is the most unique feature of humans, which makes us different from all living beings. Language development happens in stages, with the child having more and more opportunities to communicate as they age. Before learning to speak, the child must first learn to listen to and comprehend language. According to Fridman, the development of language skills starts in the intrauterine period. It starts with receptive language followed by expressive language [1]. The age at which a child starts to develop speech is zero to three years, and any issues at this age will have a significant effect on the long-term communication capabilities of the child. Language acquisition delays are frequently the earliest and most sensitive signs of specific learning disorders, pervasive developmental disorders, and intellectual disabilities. The pandemic has played an adverse role in the development of children [2]. During COVID-19, the “lock-down babies” interacted with fewer people of all ages, and they appeared to be slower in gaining skills in social communication and language. According to research, the lockdown has greatly affected the development of social and communication skills in children [2].

Gender differences in language acquisition

Early language acquisition in infants is characterized by considerable variation in timing, style, and learning strategies. The dynamic and intricate interactions between biological and environmental factors are linked to the similarities and differences in children’s acquisition processes [3]. Numerous studies have examined the role of gender in an attempt to explain this variability. These studies have found that girls have an advantage over boys in several areas related to language development, especially in the early stages of lexical development. Instead of going into more detail about possible theoretical reasons for the differences between boys and girls and the factor(s) that might cause them, most studies that have examined possible gender differences have used parental questionnaires to show different norms for boys and girls [4,5].

One popular instrument for evaluating the language development of infants and toddlers is the Language Assessment Scale Trivandrum (LEST). This is a simple screening tool used in the community to quantify language acquisition and delay, but there are no different norms on the scale based on gender. Nair et al. developed the LEST which was initially validated in young children (zero to three years old). It has been acknowledged as one of the best screening tools for identifying language and speech delays in a community context [6]. A total of 33 test items were selected from the list of items created from the pilot studies conducted among children aged zero to one year, one to two years, and two to three years. The primary goal of the scale is to make it easy for community health workers to use and comprehend to identify potential speech and language delays in children aged zero to three years and empower mothers to start speech and language stimulation at home [7,8].

In our institute, we have noticed that boys present with language delays more often than girls, especially after COVID-19 when children were locked inside their houses and the digital world took the upper hand. It is very important to determine the gender differences in the scale to emphasize the importance of separate scales for boys and girls.

Therefore, this study aims to respond to the research questions. It aims to determine if there are any gender disparities in the performance of LEST among children between zero and three years of age and to assess if there are any risk factors associated with the differences.

## Materials and methods

Study design

This cross-sectional study was conducted among 198 children (99 girls and 99 boys) between the ages of zero to three years from April 2022 to June 2023. The study was conducted at our hospital in the Department of Pediatrics, SRM Medical College and Hospital, Chennai. Informed consent was obtained from the parents.

Ethical considerations

The Local Ethics Committee of the Department of Pediatrics at SRM Medical College and Hospital approved the project (approved number: SRMIEC-ST0823-927).

Study criteria

Inclusion Criteria

All healthy children (zero to three years old) coming for regular checkups and vaccination visits were included in the study.

Exclusion Criteria

Any child with a neurological abnormality, autism spectrum disorder, an ill child, or a child who was uncooperative for testing was excluded.

Study procedure and assessments

The LEST scale was used to assess the children directly. As per the LEST scale, the first milestone is turning to sound/rattle/clap which can be assessed since birth/newborn; hence LEST scale can be used from birth until three years. For the administration of LEST, the child’s chronological age was initially determined. A vertical line was drawn by keeping a scale at the point corresponding to chronological age in months given horizontally on the X-axis. All items completed fully to the left of the scale were expected to be done by the child. If not attained by the child for that age, that item delay was assumed for the child. The scale is scored as normal if all items are done, questionable when one item is not done, and suspect and delayed when two and three or more items are not done, respectively.

Risk factors such as screen time, attention time given by parents, the primary caregiver of the child, and place of care of the child and siblings were also analyzed with a questionnaire to determine if there was any association with language development.

Screen time

According to WHO guidelines, screen time for children has been classified as no screen time below two years of age, one hour of screen time in children aged between 24 and 59 months, and less than two hours per day for children aged between five and 10 years. In our survey, children from birth to three years were asked about their screen time per day and then classified and analyzed. Children exposed to screen time independently and the mother assisting and showing the screen to newborns was also considered screen time.

Attention time

Parents were questioned regarding the time they exclusively spent interacting and engaging with their children without being distracted by any other work or factors.

Primary caregiver

Parents were questioned on who the primary caregiver for the child was and who spent the most amount of time with the child.

Primary place of care/siblings

Parents were also questioned on whether the child has any siblings, the primary place of care for the child, and if any other child was growing up in the same place.

Sample size calculation

Sample size was calculated using the following formula: $d = \mu 2-\mu 1\div r$

$d = \mu 2-\mu 1\div r n\geq \left ( 1 + r\div r \right )\left ( Z 1-\alpha /2 + Z 1-\beta \right )^{^{2}}\div d^{2}+ Z^{^{2}} 1-\alpha /2\div 2\left ( 1+r \right )$

Considering an alpha value of 0.05, a beta of 0.2, an effect size of 0.4, and a ratio (group 2/group 1) of 1, the sample size was calculated to be 200.

Statistical analysis

The numerical data obtained during the study were analyzed using SPSS statistics software version 21.0 (IBM Corp., Armonk, NY, USA). Categorical data are analyzed using frequency and the chi-square test and continuous data using an independent sample t-test. P-values <0.05 were considered statistically significant.

## Results

The study participants were analyzed and categorized based on age, gender, and risk factors. Of 198 children, 99 (50%) were girls and 99 (50%) were boys. Among 99 girls, 38 (38.4%) were aged zero to one year, 25 (25.2%) were aged one to two years, and 36 (36.3%) were aged two to three years. Among 99 boys, 41 (41.4%) were aged zero to one year, 22 (22.2%) were aged one to two years, and 36 (36.3%) were aged two to three years (Table 1).

**Table 1:** Sociodemographic characteristics of the study sample.

Sociodemographic characteristics	Girls	Boys
Age (years)		
0–1	38 (38.4%)	41 (41.4%)
1–2	25 (25.2%)	22 (22.2%)
2–3	36 (36.3%)	36 (36.3%)
Gender	99 (50%)	99 (50%)

Risk factors such as screen time, attention time given by parents, place of care for the child, primary caregiver, and siblings were assessed between boys and girls (Table 2).

**Table 2:** Risk factors for speech delay.

Risk factors	Girls	Boys
Screen time		
0–3 hours	62 (62.6%)	59 (59.5%)
4–8 hours	22 (22.2%)	40 (40.4%)
>8 hours	15 (15.1%)	12 (12.1%)
Attention time		
0–3 hours	86 (86.8%)	85 (85.8%)
4–8 hours	4 (4.0%)	8 (8.0%)
>8 hours	9 (9.0%)	6 (6.0%)
Place of care		
Home	86 (86.9%)	90 (90.9%)
Outside	13 (13.1%)	9 (9.1%)
Primary caregiver		
Mother	62 (62.6%)	67 (67.7%)
Others	37 (37.4%)	32 (32.3%)
Siblings		
Yes	51 (51.5%)	47 (47.5%)
No	48 (48.5%)	52 (52.5%)

Among the risk factors that were assessed, we found increased screen time and decreased attention time given by parents to children to be significantly associated with language delay, with a significant p-value of 0.002 and 0.020, respectively (Table 3). Children from birth to three years were asked about their screen time per day and then classified and analyzed as per WHO criteria for screen time in children. Children exposed to screen time independently and mothers assisting and showing the screen to newborns were also considered significant screen time exposure. Attention time given by parents was assessed by questioning the time they exclusively spent interacting and engaging with their child without being distracted by any other work or factors.

**Table 3:** Risk factors versus LEST interpretation. LEST = Language Assessment Scale Trivandrum

Risk factors	Normal	Questionable	Suspect	Delay	P-value
Screen time					0.002
0–3 hours	28 (84.8%)	12 (66.7%)	7 (33.3%)	15 (55.6%)	
4–8 hours	5 (15.2%)	6 (33.3%)	14 (66.7%)	12 (44.4%)	
Attention time					0.02
0–3 hours	24 (72.7%)	16 (88.9%)	21 (100.0%)	25 (92.6%)	
4–8 hours	9 (27.3%)	2 (11.1%)	0 (0.0%)	2 (7.4%)	
Place of care					0.502
Home	31 (93.9%)	15 (83.3%)	17 (81.0%)	23 (85.2%)	
Outside	2 (6.1%)	3 (16.7%)	4 (19%)	4 (14.8%)	
Primary caregiver					0.878
Mother	19 (57.6%)	12 (66.7%)	13 (61.9%)	18 (66.7%)	
Others	14 (42.4%)	6 (33.3%)	8 (38.1%)	9 (33.3%)	
Siblings					0.224
Yes	15 (45.5%)	9 (50.0%)	15 (71.4%)	12 (44.4%)	
No	18 (54.5%)	9 (50.0%)	6 (28.6%)	15 (55.6%)	

The LEST scale was interpreted and compared between boys and girls. From a sample of 99 girls, 54.1% were normal, 62.1% had a questionable language delay, 70% had a suspect delay, and 34.6% had a definite language delay. Similarly, among 99 boys, 45.9% were normal, 37.9% had a questionable language delay, 30% had a suspect delay, and 65.4% had a definite language delay, with a significant p-value of 0.003 (Table 4).

**Table 4:** Gender versus LEST interpretation. LEST = Language Assessment Scale Trivandrum

LEST	Girls	Boys
Normal	33 (54.1%)	28 (45.9%)
Questionable	18 (62.1%)	11 (37.9%)
Suspect	21 (70.0%)	9 (30.0%)
Delay	27 (34.6%)	51 (65.4%)
P-value	0.003	

## Discussion

This study examined how gender affects language acquisition, how LEST scores differ, and whether boys’ and girls’ speech delay scales should be designed differently to provide accurate results. Gender differences in LEST scores of a sample of children aged zero to three years were the focus of the first research question.

In our study on children aged between zero and three years evaluated using the LEST scale, we found that boys had a delay in acquiring language milestones compared to girls, and the difference was significant. Overall, 27 girls out of 78 delayed children (34.6%) and 51 boys out of 78 delayed children (65.4%) had language delays, with a significant p-value of 0.003.

A study conducted on the LEST scale on children aged three to six years by Singaraiah et al. found that boys had a significantly higher language delay than girls, with a male-to-female ratio of 2.2:1 [6] The male-to-female ratio in the study by Nair et al. was 1.3:1 [7]. Our study revealed a male-to-female ratio of 2:1, which was in line with the findings of Singaraiah et al. [6] and Krogh et al. [5] who used Bailey 3 scores and demonstrated gender differences in test scores; however, the pattern of the differences varied across scales and subtests.

Studies done on language assessment and speech delay have pointed toward the disadvantage of boys over girls [4,9]. The results of a systematic review by Adani et al. [4] on sex differences in early communication development demonstrated that boys are more likely to have disorders affecting the neurobiological underpinnings of complex communication systems. It appears that women acquire language and communication skills more quickly than men due to the way the female brain is functionally organized.

When language interventions were interrupted, children in a pilot study conducted by Hsu et al. [9] during COVID-19 showed a declining trend in language performance and a borderline language delay in overall language ability tests.

According to a study done by Norling et al., children’s motivation and comprehension rise when the learning environment is moved outside. Children’s ability to learn a rich and varied language depends on their ability to explore, investigate, and reflect, all of which are facilitated by outdoor activities and events [10], and COVID-19 has made it difficult for children to go out and play.

In a study done before COVID-19 between 2014 and 2016 by Rajeshwari and Lavanya [11] assessing the language delay in children between zero and three years old using the LEST scale, no significant statistical difference was observed for speech and language delay among both sexes. Our study, which was done after COVID-19, showed a significant speech delay in boys compared to girls with a significant p-value of 0.003. Further studies with a larger cohort need to be done to compare the differences in language delay before and after COVID-19 and analyze its association.

In our study, various other risk factors were analyzed to determine their association with the LEST interpretation. Increased screen time and decreased attention time given by parents to children were significantly associated with language delay, with a significant p-value of 0.002 and 0.020, respectively. On average, twice the number of normal children are exposed to less screen time compared to others. It was also noticed that the majority of parents could not spend more than three hours with their children, only 13 parents out of 200 (<10%) were able to spend more than three hours with their children, and a significant number of children in that group were found to be normal.

The major limitation of our study was that it was a cross-sectional study with a small sample size. Various studies done on other developmental scales and different age groups have analyzed various other risk factors and their association with language delay [12-15]. We suggest further longitudinal studies with a larger sample size to analyze more risk factors and validate the differences between boys and girls.

## Conclusions

Our study findings suggest that boys follow a different timeline in achieving language milestones compared to girls. These findings need to be validated with a larger study, and if found to have a significant difference, separate scales can be developed for boys and girls to assess language-acquiring skills.

## Appendices

Language assessment: boys vs. girls aged zero to three years based on the Language Assessment Scale Trivandrum

Name:

Age:

Sex:

1)         Average screen time in a day

2)         Hours of quality time spent by attendants

3)         Care given at home or at crèche?

4)         Who is the primary caregiver of the child?

5)         Has the child joined playschool?

6)         Number of siblings
